# Boosting the engraftment of subcutaneously transplanted pancreatic islets by nanofat

**DOI:** 10.1111/dom.70127

**Published:** 2025-09-24

**Authors:** Selina Wrublewsky, Caroline Bickelmann, Lea Stefanie Meßmer, Cedric Wilden, Charlotte Berhorst, Leticia Prates‐Roma, Bruce Morgan, Sandra Rother, Michael D. Menger, Matthias W. Laschke, Andrea Weinzierl, Emmanuel Ampofo

**Affiliations:** ^1^ Institute for Clinical and Experimental Surgery Saarland University, PharmaScienceHub (PSH) Homburg Germany; ^2^ Biophysics Department, Center for Human and Molecular Biology (ZHMB) Saarland University Homburg Germany; ^3^ Institute of Biochemistry, Center for Human and Molecular Biology (ZHMB), Saarland University Saarbrücken Germany; ^4^ Department of Plastic Surgery and Hand Surgery University Hospital Zurich Zurich Switzerland

**Keywords:** diabetes, insulin, islet transplantation, nanofat, vascularization

## Abstract

**Aims:**

In the therapy of type 1 diabetes mellitus, the subcutaneous space has been suggested to be a clinically preferable transplantation site for pancreatic islets due to its easy accessibility. However, its poor vascularisation capacity and, thus, challenging environment typically result in islet engraftment failure. In the present proof‐of‐principle study, we demonstrate that this problem can be overcome by nanofat, an emulsified fat derivative already used in clinical practice.

**Materials and Methods:**

The cellular composition of nanofat was assessed by immunohistochemistry. The angiogenic activity of the soluble and cellular nanofat fraction was analyzed by an angiogenic protein array, tube formation and spheroid sprouting assays. The viability and endocrine function of islets exposed to the nanofat fractions was investigated by flow cytometry, qRT‐PCR and ELISA. In vivo, islets and nanofat were co‐transplanted under the kidney capsule as well as into the subcutaneous space of diabetic animals.

**Results:**

In a panel of in vitro assays, we showed that the soluble and cellular nanofat fraction improve the viability, hormone release, and angiogenic activity of islets. The beneficial effects of these two fractions were validated in vivo in the murine diabetic kidney capsule model, as indicated by an accelerated restoration of normoglycaemia. The co‐transplantation of islets with nanofat resulted in successful islet engraftment within the subcutaneous space of diabetic mice.

**Conclusions:**

These findings demonstrate that nanofat markedly boosts the vascularisation and endocrine function of islet grafts. Hence, its co‐transplantation with pancreatic islets represents a simple, clinically feasible approach to make the subcutaneous space available for future islet transplantation.

## 1 INTRODUCTION

Subcutaneous islet transplantation is a preferential treatment option that may improve the quality of life for type 1 diabetes mellitus (T1DM) patients.^1^, ^2^ However, so far this therapeutic approach has not been widely used in clinical practice, which is mainly due to the poor vascularisation capacity of the subcutaneous space, resulting in ischaemia‐induced cell death of transplanted islets.^1^, ^2^ Accordingly, the prompt restoration of the islet blood vessel network and, thus, adequate supply of oxygen and nutrients is one of the biggest hurdles for the success of clinical subcutaneous islet transplantation.^3^, ^4^

In the last decades, tremendous efforts have been made to accelerate the revascularisation of islet grafts. For instance, several studies reported that the pre‐transplant exposure of islets to growth factors increases their endocrine and angiogenic function after transplantation.^5^, ^6^, ^7^ Furthermore, co‐transplantation of progenitor and endothelial cells has been shown to improve the revascularisation of the grafts, which, in turn, ameliorates the outcome of islet transplantation.^8^, ^9^, ^10^, ^11^ However, the stimulation of angiogenesis by growth factors as well as the differentiation of progenitor cells to endothelial cells and their reassembly into new microvessels is a time‐consuming process prone to failure. Accordingly, most of the islets die during the initial post‐transplant phase when using these approaches.^12^, ^13^

We and others have recently introduced adipose tissue‐derived microvascular fragments (MVF) as vascularization units for the improvement of islet transplantation.^14^, ^15^, ^16^ MVF are a randomized mixture of functional arterioles, capillaries and venules, which rapidly reassemble into blood‐perfused microvascular networks after transplantation.^17^, ^18^, ^19^ We could show that MVF accelerate the restoration of normoglycaemia in diabetic animals and increase the engraftment of islets in the subcutaneous space.^15^, ^16^ However, the isolation of MVF from adipose tissue involves enzymatic digestion steps, which may not be easily implemented into clinical practice due to regulatory affairs.

Nanofat can be intraoperatively generated by mechanical emulsification and filtration of adipose tissue without the use of enzymes.^20^ This derivative is widely used in the field of regenerative medicine and plastic surgery for facial rejuvenation, lipomodelling and scar repair.^21^ It is characterised by a high content of growth factors, MVF and different cell types, including progenitor cells and fibroblasts.^22^, ^23^, ^24^ The latter release glycosaminoglycans (GAG), such as hyaluronic acid (HA), which not only stimulate angiogenesis but also exert beneficial effects on islet transplantation.^25^, ^26^, ^27^, ^28^, ^29^, ^30^, ^31^

Because all these individual components markedly support the restoration of a functional microvasculature, we hypothesised that nanofat can boost the engraftment of transplanted pancreatic islets even in the challenging environment of the subcutaneous space. To test this, we first divided nanofat into a soluble nanofat fraction (SNF) and a cellular nanofat fraction (CNF) and studied their effects on the viability, hormone release, and angiogenic activity of isolated murine islets. Moreover, we validated the beneficial effects of these two fractions in the murine diabetic kidney capsule model. Finally, we co‐transplanted islets with nanofat in the subcutaneous space of diabetic mice and analysed the restoration of physiological blood glucose levels.

## 2 RESULTS

### 2.1 Isolation and composition of nanofat

**FIGURE 1:**
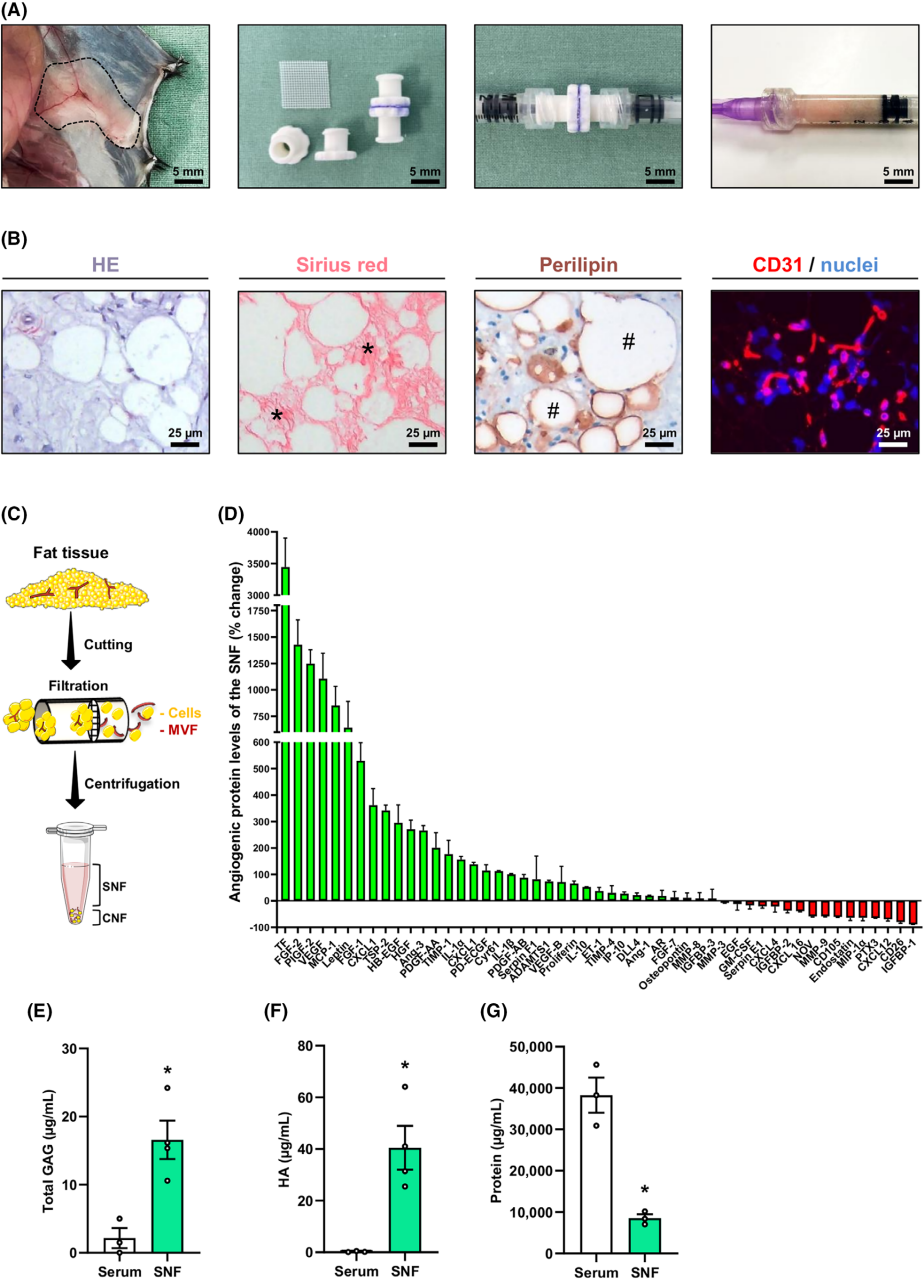
Isolation and composition of nanofat. (A) Inguinal subcutaneous adipose tissue (borders marked by broken line) for the generation of nanofat (left panel). Luer lock connectors with the cell filter (500 μm pore size) (centre left panel). Cell filter sandwiched between two Luer lock connector halves to filter the emulsified fat (centre right panel). Nanofat within a 1 mL syringe (right panel). Scale bar: 5 mm. (B) Histological HE (left panel), Sirius red (centre left panel) and perilipin (centre right panel) stainings to visualize cells and tissue structures, collagen fibres (asterisks) and adipocytes (hashtags). Immunofluorescent staining of CD31 (right panel) to visualize endothelial cells. Cell nuclei are stained with Hoechst 33342. Scale bars: 25 μm. (C) Schematic illustration of the experimental setting. Fat tissue was mechanically processed to nanofat, which was subsequently centrifuged to obtain the SNF and CNF containing MVF. (D) Quantitative analysis of angiogenic protein levels in the SNF by means of an angiogenic protein array. Serum was used as control. Data are visualized using western blotting method. Protein levels are expressed in % change of serum (*n* = 3). Mean ± SEM. (E–G) Quantitative analysis of total GAG (μg/mL) (E), total HA (μg/mL) (F) and total protein amount (μg/mL) (G) of the SNF. Serum was used as control (*n* = 3). Mean ± SEM. *p < 0.05 versus serum. CNF, cellular nanofat fraction; GAG, glycosaminoglycans; MVF, microvascular fragments; SNF, soluble nanofat fraction.

The subcutaneous adipose tissue of mice was excised and mechanically processed to nanofat by a female‐to‐female Luer lock system (Figure 1A). The obtained emulsion was then histologically stained to study its morphology and cellular composition (Figure 1B). These analyses showed that the generated nanofat contains inter alia extracellular matrix components, perilipin‐positive intact adipocytes, and CD31‐positive endothelial cells (Figure 1B).

Next, we separated nanofat in a SNF and CNF by centrifugation (Figure 1C). To assess the angiogenic activity of the SNF, we performed an angiogenic protein expression array (Figure 1D). For this purpose, serum from healthy donor mice served as a control. Of interest, we found increased levels of most of the analysed proteins in the SNF when compared to serum control by using standard western blotting (Figure 1D). For instance, we detected higher levels of the angiogenic growth factors tissue factor (TF), fibroblast growth factor (FGF)‐2, placental growth factor‐2, vascular endothelial growth factor (VEGF)‐A, heparin‐binding epidermal growth factor‐like growth factor (HB‐EGF) and hepatocyte growth factor (HGF) (Figure 1D). In addition, we measured increased levels of pro‐inflammatory cytokines, such as interleukin (IL)‐1α/β or IL‐10, which have also been shown to promote blood vessel formation.^32^, ^33^

Free GAG are crucial mediators of cellular and extracellular matrix microenvironments with the ability to specifically bind and regulate the function of ligands and receptors crucial for angiogenesis.^25^, ^34^, ^35^ Within the SNF, we found not only significantly higher levels of total soluble GAG but also of HA when compared to serum controls (Figure 1E,F). In contrast, the total protein amount in the SNF was lower (Figure 1G).

**FIGURE 2:**
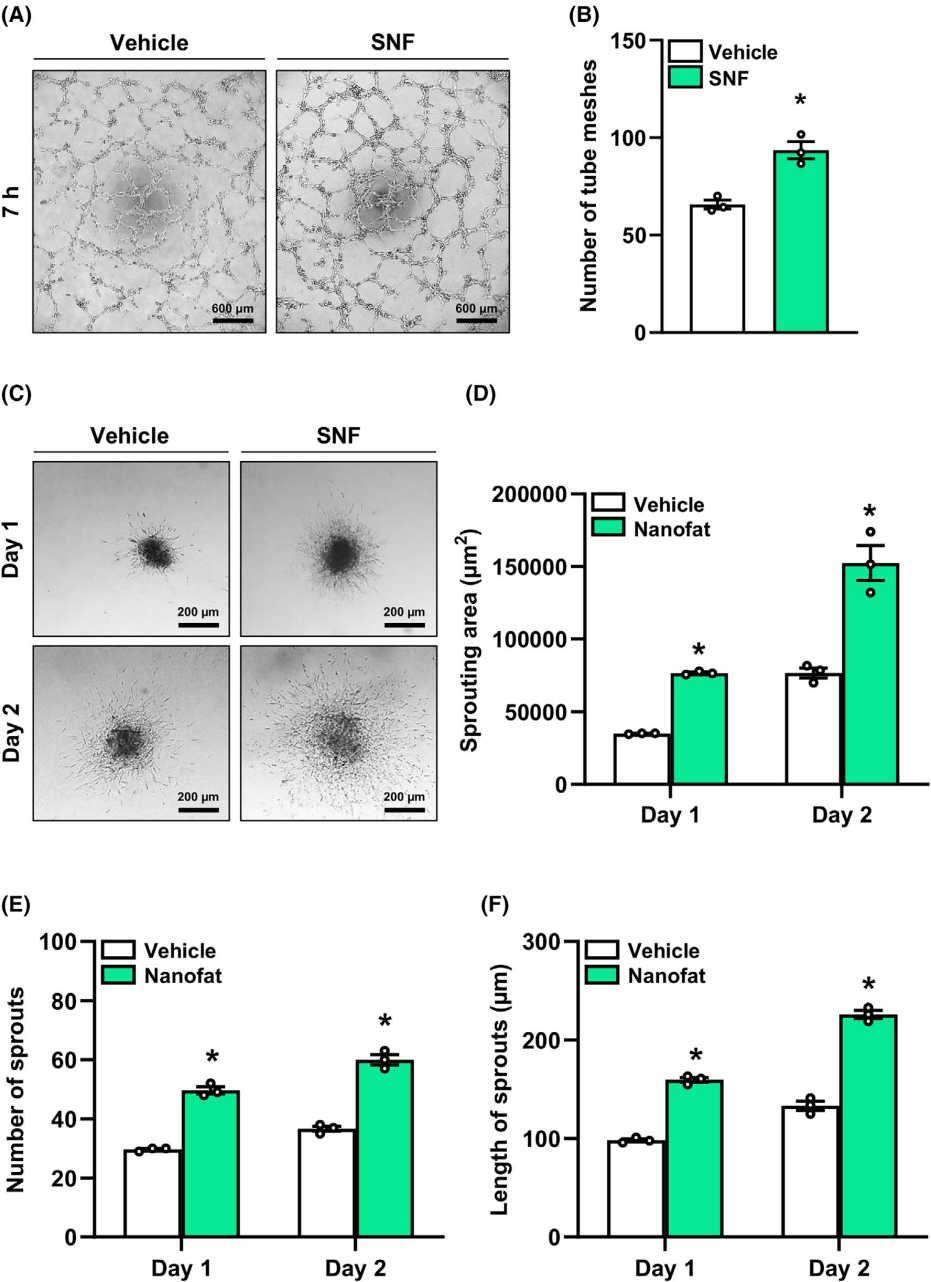
Angiogenic activity of the SNF. (A) Tube formation assays were performed with HUVEC exposed to vehicle or the SNF. The formation of vessel‐like structures was analysed 7 h after seeding (representative images). Scale bar: 600 μm. (B) Quantitative analysis of the number of tube meshes (per HPF) after 7 h (*n* = 3). Mean ± SEM. *p < 0.05 versus vehicle. (C) MVF from WT mice were fused to spheroids by means of the liquid overlay technique and cultured for 5 days. On day 0, the spheroids were embedded into a collagen matrix and exposed to vehicle or the SNF. The sprouting activity was assessed on days 1 and 2 (representative images). Scale bar: 200 μm. (D–F) Quantitative analysis of sprouting areas (D), number of sprouts (E), and length of sprouts (F) of spheroids exposed to vehicle or the SNF at the indicated days (*n* = 3). Mean ± SEM. *p < 0.05 versus vehicle. HPF, high‐power field; HUVEC, human umbilical vein endothelial cells; MVF, microvascular fragments; SNF, soluble nanofat fraction; WT, wild type.

Isolated islets contain a small fraction of intra‐islet endothelial cells, which are a functional part of revascularised islet graft.^36^ To assess whether the SNF may directly stimulate angiogenesis by activating endothelial cells, we exposed human umbilical vein endothelial cells (HUVEC) to this fraction and studied the formation of tube‐like structures. As expected, we found a higher number of tube meshes in the SNF group (Figure 2A,B). In line with these results, we additionally found a significantly higher sprouting area, sprout number and length of MVF spheroids exposed to the SNF (Figure 2C–F). Based on these results, it can be assumed that the SNF improves islet transplantation by promoting the formation of new blood vessels out of intra‐islet endothelial cells.

### 2.2 Viability, endocrine function, and angiogenic activity of islets exposed to the SNF

**FIGURE 3:**
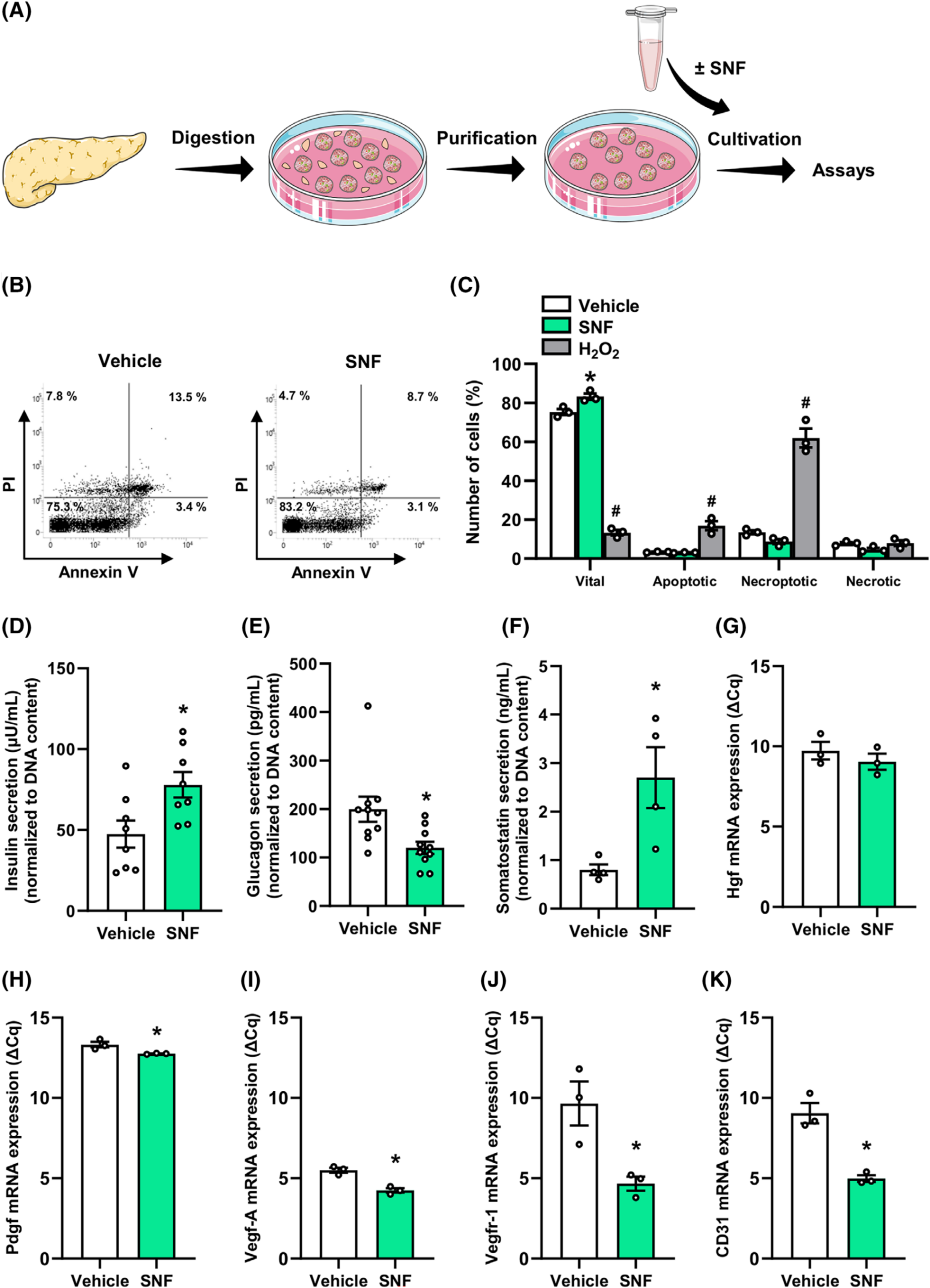
Viability and endocrine function of islets exposed to the SNF. (A) Schematic illustration of the experimental setting. Pancreatic tissue was digested, and the islets were purified to remove the exocrine tissue. Subsequently, islets were exposed to the SNF or vehicle for 24 h, and their viability and endocrine function were analysed. (B) Representative flow cytometric scatterplots of PI/annexin V‐stained cells from vehicle‐ and SNF‐treated islets. (C) Quantitative analysis of PI/annexin V‐stained cells from islets exposed to vehicle, the SNF or H_2_O_2_ (0.2%; 24 h) subdivided into necrotic, necroptotic, apoptotic and vital cells in % of total cell number (*n* = 3 each). Mean ± SEM. *p < 0.05 versus vehicle; ^#^ *p* < 0.05 versus vehicle and SNF. (D–F) Quantitative analysis of insulin secretion (μU/mL) (D), glucagon secretion (pg/mL) (E), and somatostatin (ng/mL) (F) from islets exposed to vehicle or the SNF (*n* = 4–10). Data are normalized to the total DNA content of islets. Mean ± SEM. *p < 0.05 versus vehicle. (G–K) Quantitative analysis of Hgf (G), Pdgf (H), Vegf‐A (I), Vegfr‐1 (J) and CD31 (K) mRNA expression of islets exposed to vehicle or the SNF. Data are expressed as ΔCq values (*n* = 3). Mean ± SEM. *p < 0.05 versus vehicle. Hgf, hepatocyte growth factor; Pdgf, platelet‐derived growth factor; PI, propidium iodide; SNF, soluble nanofat fraction; Vegf, vascular endothelial growth factor.

It is well known that growth factors may affect the viability and biological function of pancreatic islets.^37^, ^38^, ^39^, ^40^, ^41^, ^42^, ^43^ Therefore, we next exposed isolated murine islets to the SNF or medium as vehicle control for 24 h and studied cell survival as well as the secretion of the endocrine hormones, insulin, glucagon and somatostatin (Figure 3A). Flow cytometric analyses revealed a significantly higher number of vital cells in the SNF‐exposed islets when compared to controls (Figure 3B,C). Moreover, we found that the SNF stimulates insulin, whereas glucagon release from SNF‐exposed islets is reduced (Figure 3D–F). Of note, we also detected an increased somatostatin release, although it is known that insulin may repress somatostatin expression.^44^ Hence, we assume that the effect of the SNF on the endocrine hormone release is superior to the paracrine action of insulin, glucagon and somatostatin.

We additionally studied the gene expression of different growth factors and angiogenic receptors in SNF‐exposed islets (Figure 3G–K). Of interest, our analyses showed that the SNF stimulates the expression of Hgf, platelet‐derived growth factor (Pdgf), Vegf‐A, vascular endothelial growth factor receptor (Vegfr)‐1, and CD31 (Figure 3G–K).

### 2.3 Cellular composition and angiogenic gene expression of the CNF

**FIGURE 4:**
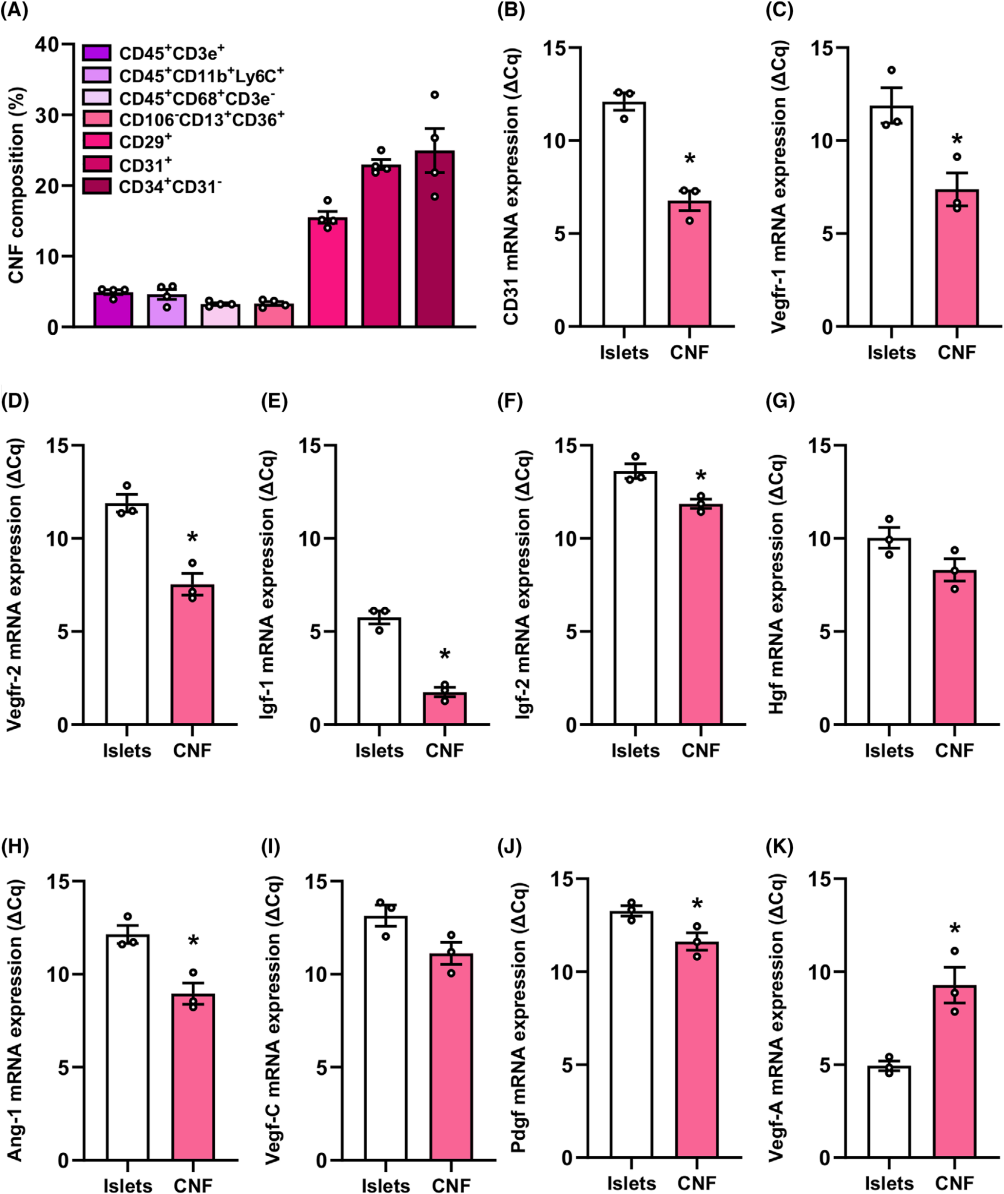
Cellular composition and angiogenic gene expression of the CNF. (A) Quantitative analysis of CD45^+^CD3e^+^, CD45^+^CD11b^+^Ly6C^+^, CD45^+^CD68^+^CD3e^−^, CD106^−^CD13^+^CD36^+^, CD29^+^, CD31^+^ and CD34^+^CD31^−^ cells within the CNF. Data are expressed in % of all cells (*n* = 4). Mean ± SEM. (B–K) Quantitative analysis of CD31 (B), Vegfr‐1 (C), Vegfr‐2 (D), Igf‐1 (E), Igf‐2 (F), Hgf (G), Ang‐1 (H), Vegf‐C (I), Pdgf (J) and Vegf‐A (K) mRNA expression of the CNF and islets. Data are expressed as ΔCq values (*n* = 3). Mean ± SEM. *p < 0.05 versus islets. CNF, cellular nanofat fraction; Hgf, hepatocyte growth factor; Pdgf, platelet‐derived growth factor; Vegf, vascular endothelial growth factor.

Next, we studied the angiogenic capacity of the CNF. The analysis of the cellular composition of this fraction showed that it contains lymphocytes (CD45^+^CD3e^+^), monocytes (CD45^+^CD11b^+^Ly6C^+^), macrophages (CD45^+^CD68^+^CD3e^−^), adipose tissue‐derived stem cells (ADSC) (CD106^−^CD13^+^CD36^+^), fibroblasts (CD29^+^), endothelial cells (CD31^+^) and pre‐adipocytes (CD34^+^CD31^−^) (Figure 4A). We further investigated the gene expression of vascular surface proteins and angiogenic factors within the CNF when compared to isolated islets. We found that CD31, Vegfr‐1/2, insulin growth factor (Igf)‐1/2, Hgf, angiopoietin (Ang)‐1, Vegf‐C and Pdgf are markedly upregulated in the CNF (Figure 4B–J). In contrast, the expression of Vegf‐A was reduced in the CNF when compared to islets (Figure 4K). The latter result is not surprising because Vegf‐A is highly expressed in β‐cells functioning as an important regulator of the islet microvasculature.^45^

### 2.4 Syngeneic transplantation of SNF‐exposed islets and syngeneic co‐transplantation of islets with the CNF under the kidney capsule of diabetic mice

**FIGURE 5:**
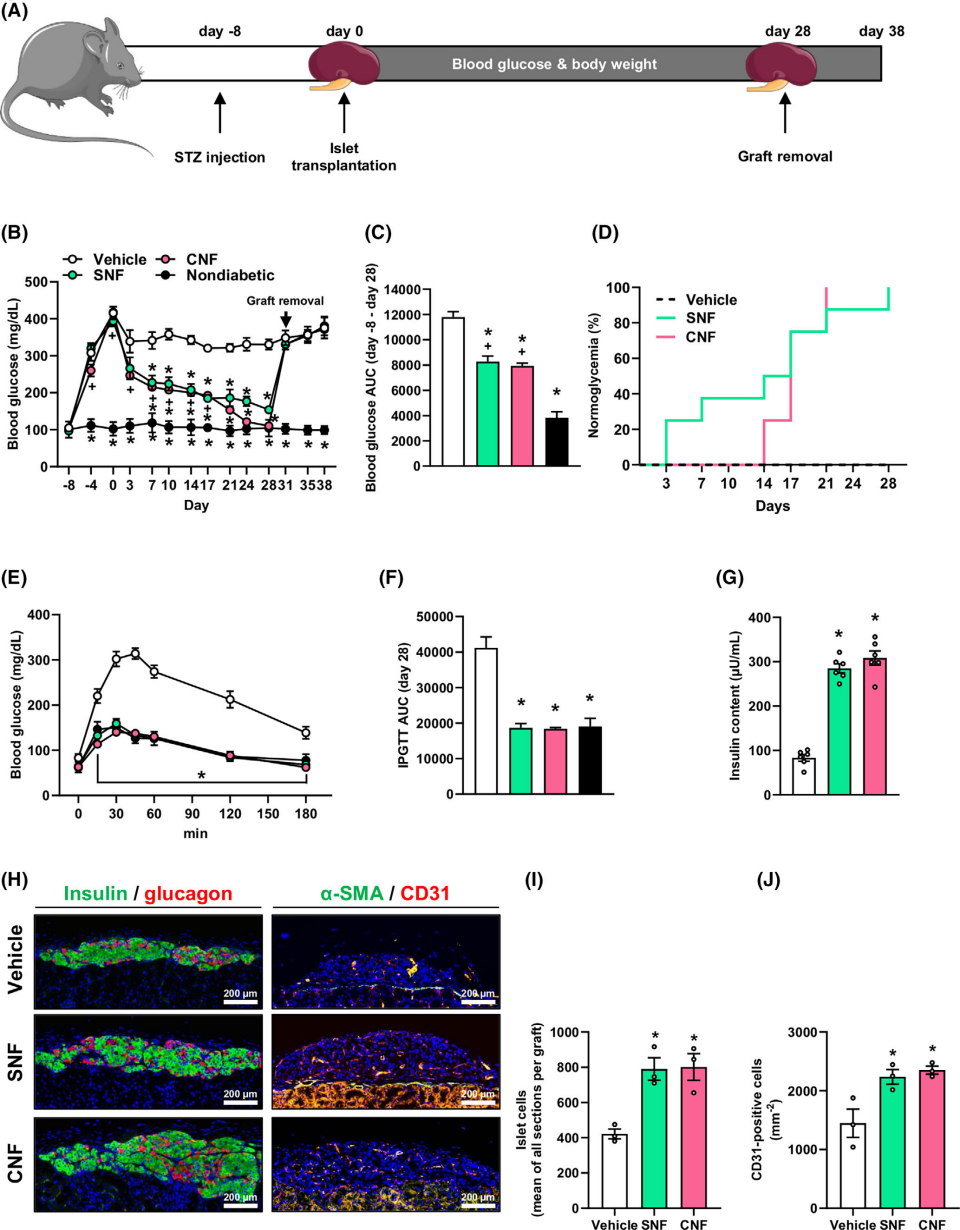
Syngeneic transplantation of SNF‐exposed islets and syngeneic co‐transplantation of islets with the CNF under the kidney capsule of diabetic mice. (A) Schematic illustration of the experimental setting. A diabetic phenotype was induced by injection of STZ 8 days prior to islet transplantation. On day 0, islets exposed to the SNF were transplanted or islets were co‐transplanted with the CNF under the left kidney capsule of diabetic mice. Blood glucose levels and body weights were measured from day −8 to day 38 twice a week. On day 28, the kidneys with the grafts were harvested. (B) Quantitative analysis of blood glucose levels (mg/mL) of diabetic mice transplanted with SNF‐exposed islets or co‐transplanted with islets and the CNF at the indicated time points. On day 28, the grafts were removed by nephrectomy (marked by arrow). Diabetic mice transplanted with vehicle‐exposed islets served as positive control and nondiabetic mice served as negative control (*n* = 8). Mean ± SEM. *p < 0.05 versus vehicle. ^+^ *p* < 0.05 versus nondiabetic. (C) AUC (day −8 to day 28) of the blood glucose levels shown in (B) (*n* = 8). Mean ± SEM. *p < 0.05 versus vehicle. ^+^ *p* < 0.05 versus nondiabetic. (D) Proportion of mice that achieved normoglycaemia after transplantation (*n* = 8). (E) Quantitative analysis of blood glucose levels (mg/dL) according to the IPGTT of diabetic mice transplanted with SNF‐exposed islets or co‐transplanted with islets and the CNF on day 28. Diabetic mice transplanted with vehicle‐exposed islets served as positive control and nondiabetic mice served as negative control (*n* = 8). Mean ± SEM. *p < 0.05 versus vehicle. (F) AUC (day 28) of the blood glucose levels shown in (E) (*n* = 8). Mean ± SEM. *p < 0.05 versus vehicle. (G) Total insulin content (μU/mL) of the removed grafts (*n* = 6). Mean ± SEM. *p < 0.05 versus vehicle. (H) Immunofluorescent stainings of insulin/glucagon and α‐SMA/CD31 within grafts of the different groups. Scale bar: 200 μm. (I) Quantitative analysis of islet cells (mean of all sections per graft) within the different groups. Mean ± SEM. *p < 0.05 versus vehicle. (J) Quantitative analysis of CD31‐positive cells (mm^−2^) within the different groups. Mean ± SEM. *p < 0.05 versus vehicle. AUC, area under the curve; CNF, cellular nanofat fraction; IPGTT, intraperitoneal glucose tolerance test; SNF, soluble nanofat fraction; STZ, streptozotocin.

To validate our in vitro findings in an in vivo setting, we exposed 250 isolated murine islets to the SNF or medium as vehicle control (positive control) for 24 h and subsequently transplanted them under the kidney capsule of streptozotocin (STZ)‐induced diabetic mice. In an additional group, islets were co‐transplanted with the CNF. Nondiabetic animals served as negative control. Blood glucose levels and body weights of the animals were determined over 28 days (Figure 5A). At the end of the observation period, the grafts were removed to exclude the spontaneous recovery of pancreatic islets in STZ‐treated mice. The body weights of nondiabetic animals as well as animals in the diabetic SNF or CNF groups did not markedly differ during the entire observation period (Supplementary Figure 1A,B). Moreover, the transplantation of a critical number of 250 islets alone did not lead to normoglycaemia. Of interest, we detected a rapid decrease in blood glucose levels of animals in the SNF or CNF groups after transplantation (Figure 5B). Accordingly, the area under the curve (AUC) was significantly reduced (Figure 5C). Furthermore, we observed that the SNF group achieved euglycaemia faster than the CNF group (Figure 5D). This effect could be due to the pretreatment of islets with the SNF, which enhances insulin secretion and, thus, may promote islet engraftment. We found lower blood glucose levels of mice in the SNF and CNF groups that underwent an intraperitoneal glucose tolerance test (IPGTT) on day 28 after transplantation (Figure 5E,F). At the end of the in vivo experiments, the kidneys were harvested, and the insulin content as well as the cellular composition of the grafts were assessed (Figure 5G,H and Supplementary Figure 2). As expected, we determined a significantly higher insulin content in the SNF and CNF groups when compared to controls (Figure 5G). Moreover, the grafts in these groups also exhibited more islet cells (Figure 5H,I) and an increased microvessel density as shown by higher numbers of CD31‐positive cells (Figure 5H,J).

### 2.5 Syngeneic co‐transplantation of islets with nanofat into the subcutaneous space of diabetic mice

**FIGURE 6:**
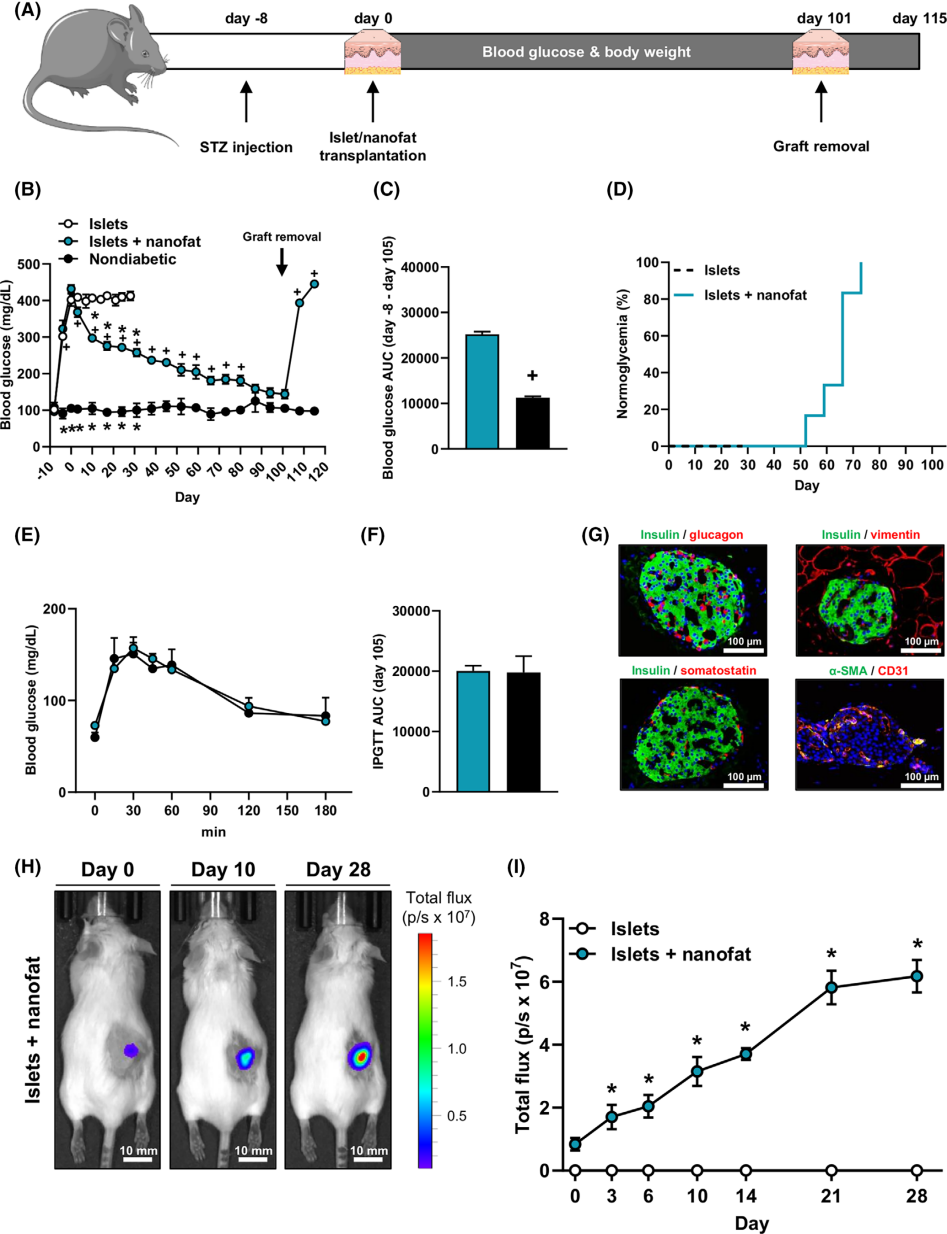
Syngeneic co‐transplantation of islets with nanofat into the subcutaneous space. (A) Schematic illustration of the experimental setting. A diabetic phenotype was induced in mice by injection of STZ 8 days prior to transplantation of islets into the subcutaneous space. Blood glucose levels and body weights were measured from day −8 to day 115 twice a week. On day 101, the subcutaneous tissue with the grafts was harvested and blood samples were collected. (B) Quantitative analysis of blood glucose levels (mg/dL) of diabetic mice transplanted with islets and nanofat. On day 101, the grafts were removed (marked by arrow). Diabetic mice transplanted with islets served as positive control and nondiabetic mice served as negative control (*n* = 6). Mean ± SEM. *p < 0.05 versus vehicle. ^+^ *p* < 0.05 versus nondiabetic. (C) AUC (day −8 to day 101) of the blood glucose levels shown in (B). Mean ± SEM (*n* = 6). ^+^ *p* < 0.05 versus nondiabetic. (D) Proportion of mice that achieved normoglycaemia after transplantation (*n* = 6). (E) Quantitative analysis of blood glucose levels (mg/dL) according to the IPGTT of diabetic mice co‐transplanted with islets and nanofat on day 101. Nondiabetic animals served as negative control (*n* = 6). Mean ± SEM. (F) AUC (day 101) of the blood glucose levels shown in (E) (*n* = 6). Mean ± SEM. (G) Immunofluorescent stainings of insulin/glucagon, insulin/vimentin, insulin/somatostatin and α‐SMA/CD31 within grafts of mice co‐transplanted with islets and nanofat on day 101. Scale bar: 100 μm. (H) Bioluminescent images of diabetic mice co‐transplanted with islets from a WT mouse and nanofat from a luciferase‐positive mouse. Scale bar: 10 mm. (I) Quantitative analysis of the total flux (p/s × 10^7^) of diabetic mice transplanted with islets or islets with nanofat (*n* = 5). Mean ± SEM. *p < 0.05 versus islets. AUC, AUC, area under the curve; IPGTT, intraperitoneal glucose tolerance test; STZ, streptozotocin; WT, wild type.

Finally, we tested whether nanofat improves the outcome of subcutaneous islet transplantation. This transplantation site exhibits a poor vascularisation capacity when compared to the kidney capsule.^46^, ^47^ Accordingly, the revascularisation process takes more time, which is why more islets have to be transplanted. For this purpose, a critical number of 500 islets was co‐transplanted with nanofat into the subcutaneous space of STZ‐induced diabetic mice. Diabetic animals receiving the identical critical number of 500 islets alone served as positive control, whereas nondiabetic animals served as negative control. We followed graft function for >100 days (Figure 6A). During the entire observation period, we did not detect any differences in the body weights between the different groups (Supplementary Figure 3A,B). Notably, co‐transplantation of islets with nanofat markedly reduced hyperglycaemia (Figure 6B,C). Accordingly, we measured physiological blood glucose levels in all animals of the co‐transplantation group at the end of the observation period (Figure 6D). In addition, an IPGTT on day 101 after transplantation showed that mice receiving islets and nanofat exhibit similar blood glucose levels when compared to nondiabetic animals (Figure 6E,F).

Histochemical analyses of the grafts demonstrated that they contain β‐, α‐, δ‐ and endothelial cells, as shown by positive insulin, glucagon, somatostatin and α‐SMA/CD31 stainings (Figure 6G and Supplementary Figure 4). To analyse whether the co‐transplanted nanofat survived the transplantation process and, thus, could continuously promote the engraftment of the islets, we isolated nanofat in additional experiments from luciferase‐positive mice (Supplementary Figure 5A–D). This transgenic nanofat was then co‐transplanted with islets from wild‐type donors into the subcutaneous space of luciferase‐negative mice. We detected an increasing luciferase signal at the transplantation site during the first 28 days (Figure 6H,I), indicating that the nanofat survives the initial, critical post‐transplantation phase.

## 3 DISCUSSION

Nanofat is an autologous fat derivative that has been well established in clinical use and is generated by mechanical emulsification followed by filtration to obtain a liquid suspension.^20^ This suspension can be divided into two parts: the CNF, which is characterised by a high number of MVF and progenitor cells, such as ADSC, and the SNF containing growth factors, cytokines and chemokines.^22^, ^23^, ^24^ Accordingly, nanofat may ideally support the vascularisation of transplanted islets.

To test this hypothesis in the present study, we analysed the effects of the SNF on the viability of isolated murine islets. It is known that ADSC are superior to other progenitor cells in secreting paracrine molecules stimulating cell proliferation.^48^ Moreover, several groups reported that ADSC co‐cultured with islets release factors enhancing islet viability.^49^, ^50^, ^51^ In line with these findings, we detected a significantly higher number of vital cells in islets exposed to the SNF.

We additionally performed several well‐established in vitro angiogenesis assays to assess the angiogenic activity of the SNF. We found that the SNF indeed promotes the formation of tube‐like structures from HUVEC and the sprouting of MVF spheroids. To elucidate the underlying mechanisms, we measured the expression of multiple angiogenic factors by means of a protein array. We detected high levels of angiogenic factors, such as TF, FGF‐1/2/7, VEGF, HB‐EGF, and IL‐1α/β in the SNF when compared to serum controls. In contrast, we also detected low levels of angiogenic factors such as pentraxin (PTX) 3, which may have beneficial effects on islet survival. In fact, Kim et al.^52^ identified the pivotal role of PTX3 in the pathogenesis of hyperglycaemia, pancreatic endoplasmic reticulum stress, and β‐cell apoptosis. They could show that not only exogenous PTX3 induces β‐cell apoptosis in mouse islets but also the loss of PTX3 prevents STZ–induced pancreatic injury. Accordingly, it can be assumed that the angiogenic activity of the SNF is superior to the physiologically endogenous angiogenic activity of serum. In addition, we studied for the first time the levels of free GAG in the SNF. GAG are important extracellular matrix molecules modulating inflammation and angiogenesis.^35^ We and others have already reported that HA stimulates angiogenesis by different signalling pathways, such as transforming growth factor or VEGF.^28^, ^29^, ^53^ Moreover, beneficial effects of HA on islet transplantation have been described in the past.^30^, ^31^ Interestingly, we found high levels of HA in the SNF. Taken together, we assume that all these factors orchestrate the vascularisation process of transplanted islets by stimulation of angiogenic processes in islet cells as well as in the host tissue.

We further studied the effect of the SNF on the endocrine function of isolated islets. We detected an improved glycometabolic control of islets exposed to the SNF, as shown by a higher glucose‐stimulated insulin secretion as well as a decreased glucagon secretion. The SNF itself not only contained growth factors but also stimulated the expression of growth factors in islet cells. For instance, we found high levels of HGF, which has been previously shown to stimulate insulin release,^37^, ^38^ in SNF‐exposed islets. In addition, we measured elevated levels of FGF‐1/2. The positive functions of both hormones are well described. FGF‐2 influences the formation and development of pancreatic islets,^42^ while FGF‐1 increases cell survival and insulin secretion.^43^ Based on these findings, we speculated that the SNF improves the engraftment of transplanted islets. In line with this view, our in vivo experiments in the kidney capsule model showed that SNF‐exposed islets markedly accelerate the restoration of physiological blood glycose levels in diabetic animals when compared to controls.

Nanofat contains many progenitor cells that promote tissue regeneration through both angiogenic factor secretion and multi‐lineage cell differentiation.^54^ In addition, nanofat is a rich source of MVF, which has been shown to accelerate the vascularization of transplanted islets.^15^, ^16^ Therefore, we additionally investigated in the kidney capsule model whether the angiogenic capacity of the CNF ameliorates islet transplantation. As expected, the co‐transplantation of islets with the CNF enhanced their engraftment. This may be particularly due to the ADSC within the CNF. Ren et al.^55^ reported that ADSC can rescue transplanted islets from hypoxia‐induced cell death by triggering angiogenesis and suppressing inflammation. Additional analyses revealed that this is caused by an enhanced secretion of factors, such as HGF and Ang‐1, from the ADSC.^55^ Of note, we also detected high levels of these two factors in the SNF. Furthermore, our immunohistochemical analyses proved the presence of MVF in nanofat. Accordingly, we also measured a high number of endothelial cells within the CNF by flow cytometry. Therefore, it is conceivable that both ADSC and MVF of the CNF support the engraftment of transplanted islets.

Clinical islet transplantation can be performed minimal‐invasively by an intraportal infusion of islets, which provides an adequate oxygen and nutrient supply.^56^ A potential complication of this procedure is the instant blood‐mediated inflammatory reaction (IBMIR). This is characterised by platelet aggregation and activation of the clotting cascade resulting in a massive islet loss after transplantation.^57^ To circumvent IBMIR, intensive efforts have been made to identify favourable extrahepatic sites. The subcutaneous space has appealing potential for clinical islet transplantation due to its easy accessibility. However, the poor vascularisation capacity and low tissue oxygen tension are major problems of this transplantation site.^46^, ^47^ In the past, tremendous efforts have been made to overcome this problem. For instance, Aghazadeh et al.^14^ demonstrated that the co‐transplantation of MVF and stem cell‐derived β‐cells into a subcutaneous pocket maintains long‐term normoglycaemia. Moreover, we have already reported that the subcutaneous co‐transplantation of intact islets with MVF sufficiently reverses hyperglycaemia in STZ‐treated animals.^16^ However, MVF are currently not broadly used in clinical practice as vascularisation units because their isolation by means of enzymatic digestion of adipose tissue is not yet intraoperatively feasible due to the lack of good manufacturing practice‐conform devices.^20^, ^58^ Our novel results now indicate that nanofat represents an attractive alternative, especially as adipose tissue is abundantly available in most patients with minimal donor site morbidity. In fact, we clearly showed that the co‐transplantation of a critical number of 500 islets with this MVF‐containing fat derivative into the subcutaneous space restores normoglycaemia, whereas the transplantation of 500 islets alone did not lead to physiological blood glucose levels in diabetic mice.

Taken together, the present proof‐of‐principle study demonstrates for the first time that nanofat markedly boosts the engraftment of pancreatic islets. Clinical islet transplantation is one of the safest and least invasive transplant procedures and has been performed for the last three decades.^56^ In addition, nanofat is successfully used for lipomodelling and scar repair.^21^ Accordingly, the herein developed strategy of the co‐transplantation of islets with nanofat may be transferable into clinical practice. In fact, it is conceivable to isolate nanofat in an intraoperative setting from the T1DM patient. This autologous approach would prevent an immune response after transplantation caused by the cellular and soluble part of the nanofat and, thus, falls under the minimal manipulation norms of the FDA guidelines. The nanofat is then co‐transplanted with allogenic islets, which typically requires a specific immunosuppression regimen.^59^ Our novel findings now open the door for a completely new scope of nanofat in the therapy of T1DM, that is, improving the outcome of subcutaneous islet transplantation.

## 4 METHODS

### 4.1 Reagents

Collagenase NB 8 broad range was purchased from Nordmark Biochemicals (Uetersen, Germany). Roswell Park Memorial Institute (RPMI)‐1640, Dulbecco's modified Eagle medium (DMEM), bovine serum albumin (BSA), the bicinchoninic acid (BCA) protein assay and mouse insulin enzyme‐linked immunosorbent assay (ELISA) kit were purchased from Thermo Fisher Scientific (Karlsruhe, Germany). Endothelial Cell Basal Medium (ECBM) was purchased from PromoCell (Heidelberg, Germany). Glucagon ELISA kit, proteome profiler mouse angiogenesis array kit, recombinant aggrecan and biotinylated aggrecan were purchased from R&D systems (Minneapolis, USA). Somatostatin ELISA kit was purchased from Phoenix Pharmaceuticals (Burlingame, USA). Hoechst 33342, neutral red solution, penicillin, collagenase IAS, Sirius red, chondroitin sulphate C and STZ were purchased from Sigma‐Aldrich (Taufkirchen, Germany). HepatoQuick® and annexin‐V‐FLUOS staining kit were purchased from Roche (Basel, Switzerland). Propidium iodide (PI) was purchased from BD Biosciences (San Jose, USA). Ketamine (Ursotamin®) was purchased from Serumwerke Bernburg (Bernburg, Germany). Xylazine (Rompun®) was purchased from Bayer (Leverkusen, Germany). Carprofen (Rimadyl®) was purchased from Zoetis Deutschland GmbH (Berlin, Germany). IVISbrite d‐luciferin potassium salt bioluminescent substrate was purchased from Revvity (Hamburg, Germany). Accutase was purchased from BioLegend (Koblenz, Germany). Fetal calf serum (FCS) was purchased from Biochrom GmbH (Berlin, Germany). Collagen was purchased from Advanced BioMatrix (Carlsbad, USA). Cell lysis reagent QIAzol was purchased from Qiagen (Hilden, Germany). The qScriber cDNA synthesis kit and ORA SEE qPCR Green ROX L Mix were purchased from HighQu (Kraichtal, Germany). Matrigel was purchased from Corning (Wiesbaden, Germany). Haematoxylin and eosin (HE) were purchased from Carl Roth GmbH (Karlsruhe, Germany).

### 4.2 Antibodies

The CD3e‐FITC (22150033) antibody was purchased from Immunotools (Friesoythe, Germany). The CD11b‐FITC (11‐0112‐85), anti‐rat IgG Alexa Fluor 488 (A‐21434) and anti‐rabbit IgG Alexa Fluor 555 (A‐21429) antibodies were purchased from Thermo Fisher Scientific (Karlsruhe, Germany). The CD45‐PerCP (557235), CD106‐FITC (553332), CD68‐PE (566386), Ly6C‐PE (560593), CD36‐APC (562744) and CD31‐FITC (553372) antibodies were purchased from BD Biosciences (San Jose, USA). The CD34‐PE (128609), CD13‐PE (164003), CD36‐APC (102611) and CD29‐FITC (102205) antibodies were purchased from BioLegend (Koblenz, Germany). The anti‐CD31 antibody (DIA310) was purchased from Dianova (Hamburg, Germany). The anti‐insulin (15848‐1‐AP) and anti‐glucagon (67286‐1‐lg) antibodies were purchased from Proteintech (Rosement, USA). The anti‐somatostatin (Ab30788) antibody was purchased from Abcam (Cambridge, UK). The anti‐perilipin antibody was purchased from Cell Signaling (Danvers, USA).

### 4.3 Cell culture

HUVEC were cultivated in ECBM (100 U/mL penicillin and 0.1 mg/mL streptomycin) at 37°C under a humidified 95% to 5% (v/v) mixture of air and CO_2_. The cells were passaged at a split ratio of 1:3 after reaching confluence.

### 4.4 Tube formation assay

For the tube formation assay, HUVEC were seeded in a 96‐well plate (1.5 × 10^4^ cells per well), which contained 50 μL matrigel per well. The cells were exposed to the SNF or the same amount of cell culture medium as a control (vehicle). Phase‐contrast light micrographs were taken after 7 h. Tube formation was quantified by measuring the number of tube meshes (i.e., areas completely surrounded by endothelial tubes) per high‐power field (HPF) using the ImageJ software (U.S. National Institutes of Health [NIH]).

### 4.5 SNF matrix characterization

A dimethylmethylene blue (DMMB) assay was used to quantify the amount of sulphated GAG in the SNF when compared to serum. Chondroitin sulphate C was used as the GAG standard for calibration. The absorbance of the formed DMMB dye/GAG complex was measured at 525 nm. In addition, the amount of the non‐sulphated GAG HA was analysed in diluted SNF samples via sandwich ELISA following the manufacturer's protocol (BioTechne, Wiesbaden, Germany). For this purpose, recombinant aggrecan served as the capture reagent for HA and biotinylated aggrecan was used for HA detection. Furthermore, the protein content within the SNF samples was determined with the BCA protein assay according to the manufacturer's protocol using BSA as the reference protein for calibration. The absorbance of the reaction product was measured at 565 nm.

### 4.6 Animals

C57BL/6J or FVB‐Tg(CAG‐luc,‐GFP)L2G85Chco/J mice with a body weight of ~30 g served as donors for MVF isolation and for nanofat generation. C57BL/6J mice with a body weight of 25–28 g served as donors for islet isolation. Diabetes was induced in male C57BL/6J mice with a body weight of 24–28 g. All animals were maintained on a standard 12/12 h day/night cycle. Water and standard pellet chow (Altromin, Lage, Germany) were provided ad libitum.

The experiments were performed according to the German legislation on protection of animals and the NIH Guide for the Care and Use of Laboratory Animals (Institute of Laboratory Animal Resources, National Research Council, Washington DC, USA). They were approved by the local governmental animal protection committee (permission number: 18/2017 and 41/2019).

### 4.7 Isolation of pancreatic islets

Mice were anaesthetised by intraperitoneal (i.p.) injection of ketamine (100 mg/kg body weight) and xylazine (12 mg/kg body weight). Following cervical dislocation and midline laparotomy, the pancreatic duct was injected with 1 mg/mL collagenase NB 8 containing 25 μL/mL neutral red solution. Neutral red has no negative impact on islet viability and is commonly used to isolate pancreatic islets.^60^ The pancreas was excised and further digested by collagenase NB 8 (1 mg/mL). After washing with phosphate buffered saline (PBS) containing 10% FCS to inactivate the collagenase, the islets were purified by hand picking. Islets were then transferred in RPMI‐1640 (supplemented with 10% [v/v] FCS, 100 U/mL penicillin and 0.1 mg/mL streptomycin).

### 4.8 Nanofat generation

The inguinal subcutaneous adipose tissue of anaesthetised C57BL/6J or FVB‐Tg(CAG‐luc,‐GFP)L2G85Chco/J mice was excised, taking care not to include the inguinal lymph node. The tissue was then minced by means of a histology tissue cutter (McIlwain Tissue Chopper, CLE Co. Ltd., Gomshall, UK) to produce fat fragments of an identical volume (~ 1 × 1 × 1 mm), which were rinsed in 0.9% NaCl solution. For emulsification, the fat was shuffled between two syringes using three female‐to‐female Luer lock connectors with descending internal diameters of 2.4, 1.4 and 1.2 mm and 30 passes per connector. In a last step, the fat was passed through a cell filter with 500 μm pore size to remove any larger remaining fat particles or debris. The generated nanofat was separated into a SNF and CNF by centrifugation at 10000*g* for 10 min.

### 4.9 Angiogenic protein expression array

The expression of 51 angiogenesis‐related proteins of the SNF and murine serum (as control) was analysed by means of a membrane‐based sandwich immunoassay (proteome profiler mouse angiogenesis array kit). For this purpose, 1 mL of the SNF or serum was incubated with the array membrane according to the manufacturer's instructions. The proteins were visualised by means of chemiluminescent detection reagents in a Chemocam device (Intas, Göttingen, Germany). The intensity of the measured signals was quantified using the ImageJ software.

### 4.10 Isolation of MVF and spheroid sprouting assay

Mice were anaesthetised and euthanised by cervical dislocation. Subsequently, MVF were isolated by mechanical and enzymatic digestion (collagenase IAS) of the isolated visceral fat pads, as described previously in detail.^19^ After isolation, the MVF were cultivated in DMEM (10% [v/v] FCS, 100 U/mL penicillin and 0.1 mg/mL streptomycin) at 37°C and 5% CO_2_. MVF spheroids were generated by the liquid overlay technique in a 96‐well plate covered with 1% agarose. For this purpose, 750 MVF were seeded per well and cultivated for 5 days to allow the formation of a spheroid at 37°C under a humidified 95% to 5% (v/v) mixture of air and CO_2_. After 5 days, the spheroids were harvested. The angiogenic activity of the MVF spheroids was determined by means of a sprouting assay. MVF spheroids were collected and resuspended in a collagen solution to transfer them into prewarmed 24‐well plates.^61^ After 45 min, the collagen gel was covered with DMEM (10% (v/v) FCS, 100 U/mL penicillin and 0.1 mg/mL streptomycin) and the spheroids were incubated for 2 days at 37°C and 5% CO_2_ for daily analyses. The sprouting was visualised by a BX60F microscope (Olympus, Hamburg, Germany) and assessed by measuring the sprouting area, number and length of sprouts with the FIJI software (NIH).

### 4.11 Flow cytometry

The CNF was dispersed into single cells by accutase. The cells were washed in PBS and incubated with the indicated antibodies for 30 min at room temperature. The numbers of positive cells for the indicated antibodies were determined by a FACSLyric flow cytometry system (BD Biosciences).

For PI/annexin V stainings, isolated islets were dispersed into single cells by trypsin. Subsequently, the cells were washed in PBS, resuspended in the incubation buffer and stained for 15 min with PI and annexin V according to the manufacturer's protocol. The stained cells were analysed by flow cytometry using a FACSLyric flow cytometry system (BD Biosciences) and the fractions of vital, apoptotic, necrotic as well as necroptotic cells were given in % of all measured cells.

### 4.12 Quantitative real‐time polymerase chain reaction (qRT‐PCR)

Total RNA from islets exposed to the SNF or vehicle as well as from the CNF was isolated using QIAzol lysis reagent (Qiagen, Hilden, Germany). The corresponding cDNA was synthesized from 1 μg of total RNA by QuantiNova Reverse Transcription Kit (Qiagen) according to the manufacturer's instructions. ORA qPCR Green ROX L Mix (highQu) was used for qRT‐PCR. Data analysis was performed by the MiniOpticon Real‐Time PCR System (Bio‐Rad, Feldkirchen, Germany). Murine GAPDH served as an internal control for mRNA detection.

Forward and reverse primers were used in a concentration of 700 nM solved in RNase/DNase‐free H_2_O. Primer sequences for qPCR were coded as follows: Mouse Hgf forward 5′‐GCTGGTCTATGGTCCTGAAG‐3′, reverse 5′‐TGATCAATCCAGTGTAGCCC‐3′; mouse Pdgf forward 5′‐CCTTAGTGGTCCTTACCGTC‐3′, reverse 5′‐TTCCATCGGATCTCATAGCG‐3′; mouse Vegf‐A forward 5′‐GCTGTACCTCCACCATGCCAAG‐3′, reverse 5′‐CGCACTCCAGGGCTTCATCG‐3′; mouse Vegf‐C forward 5′‐CTGATGTCTGTCCTGTACCC‐3′, reverse 5′‐TCCCCACATCTATACACACC‐3′; mouse Vegfr‐1 forward 5′‐AAGACGGTTAGCACATTGGT‐3′, reverse 5′‐TCGGCACATCTGTGACATAA‐3′; mouse Vegfr‐2 forward 5′‐CCTACCTCACCTGTTTCCTG‐3′, reverse 5′‐TCTGGCTGTCATCTGGGAT‐3′; mouse Igf‐1 forward 5′‐GATGCTCTTCAGTTCGTGTG‐3′, reverse 5′‐CACAGCTCCGGAAGCAACAC‐3′; mouse Igf‐2 forward 5′‐CGTACTTCCGGACGACTTC‐3′, reverse 5′‐TGGGTGGTAACACGATCA‐3′; mouse Ang‐1 forward 5′‐CATTCTTCGCTGCCATTCTG‐3′, reverse 5′‐GCACATTGCCCATGTTGAATC‐3′; mouse CD31 forward 5′‐CACAGAAGTGGAAGTGTCCT‐3′, reverse 5′‐ACCTTCCGGATCTCACTGT‐3′; mouse GAPDH forward 5′‐TGACCTCAACTACATGGTCTAC‐3′, reverse 5′‐CTTCCCATTCTCGGCCTTG‐3′.

### 4.13 Enzyme‐linked immunosorbent assay

The amount of secreted insulin was measured by a mouse insulin ELISA kit. For this purpose, 10 isolated islets were washed with Krebs Ringer Buffer (KRB) (115 mM NaCl, 4.7 mM KCl, 1.28 mM CaCl_2_, 1.2 mM MgSO_4_, 0.1% BSA) and incubated for 1 h at 37°C and 5% CO_2_. The supernatants were discarded, and the islets were incubated for 30 min in KRB containing 16.5 mM glucose. The supernatants were collected, and the amount of secreted insulin was determined by using an insulin ELISA kit according to the manufacturer's protocol.

The amount of secreted glucagon was measured by a glucagon ELISA kit. For this purpose, 20 islets were cultivated in KRB buffer (115 mM NaCl, 4.7 mM KCl, 1.28 mM CaCl_2_, 1.2 mM MgSO_4_, 0.1% BSA, 25 mM glucose) for 1 h at 37°C and 5% CO_2_. Subsequently, the buffer was removed, and the islets were cultivated in KRB buffer containing 0.5 mM glucose for 2 h. The supernatants were collected, and the amount of secreted glucagon was determined by using a glucagon ELISA kit according to the manufacturer's protocol.

The amount of secreted somatostatin was measured by a somatostatin ELISA kit. For this purpose, 20 islets were cultivated in KRB buffer (140 mM NaCl, 3.6 mM KCl, 2.6 mM CaCl_2_·H_2_O, 0.5 mM MgSO_4_·7H_2_O, 0.5 mM NaH_2_PO_4_, 2 mM NaHCO_3_, 5 mM HEPES, 1 mM glucose) for 30 min at 37°C and 5% CO_2_. Subsequently, the buffer was removed, and the islets were cultivated in KRB buffer (70 mM NaCl, 70 mM KCl, 2.6 mM CaCl_2_H_2_O, 0.5 mM MgSO_4_·7H_2_O, 0.5 mM NaH_2_PO_4_, 2 mM NaHCO_3_, 5 mM HEPES, 20 mM glucose) for 2 h. The supernatants were collected, and the amount of secreted somatostatin was determined by using a somatostatin ELISA kit according to the manufacturer's protocol.

### 4.14 Diabetes induction and syngeneic islet transplantation

Diabetic phenotypes were induced by a single i.p. injection of 180 mg/kg STZ 8 days prior to islet transplantation. Body weights and non‐fasting blood glucose levels of STZ‐injected mice were measured twice a week during the entire observation period of 38 or 115 days. Blood samples were taken from the tail vein and analysed by a portable blood glucose monitoring system (GL50; Breuer, Ulm, Germany). Mice with a non‐fasting blood glucose level ≥ 350 mg/dL served as diabetic recipients for islet transplantation.

To exclude that these size effects influence the outcome of islet transplantation between the groups, we collected islets from multiple donors, pooled them, and then distributed them to the different recipients. In a first set of experiments, 250 SNF‐exposed islets (volume of 10 μL SNF) or 250 islets with the CNF (volume of 10 μL saline) were injected under the left kidney capsule of diabetic mice. For this, a small nick was made in the kidney capsule with the bevel of a 10 μL Hamilton syringe over the inferior renal pole. Islets were then deposited under the capsule through the nick towards the superior pole of the kidney. The kidney was returned to the retroperitoneal space and the incisions were closed. Diabetic animals receiving 250 vehicle‐exposed islets served as positive controls, whereas nondiabetic animals, which did not receive islets and CNF, were used as negative controls.

In another set of experiments, a mixture of 500 islets and 100 μL nanofat was injected into the subcutaneous space of diabetic mice. For this purpose, the nanofat was injected with a 24 G needle (Braun, Melsungen, Germany) in the subcutaneous space on the medial dorsum of the animals. After removing the needle, the islets were transplanted by means of a 10‐μL Hamilton syringe into the nanofat depot. Diabetic animals receiving 500 islets alone served as positive controls. Nondiabetic animals, which did not receive islets, were used as negative controls. Normoglycaemia was defined by blood glucose levels ≤ 200 mg/dL.

To confirm graft‐dependent normoglycaemia, the islet transplants were explanted either by nephrectomy or subcutaneous graft excision. For this purpose, the graft‐bearing kidney was exposed, and the renal vessels and the ureter were ligated at the pedicle. Thereafter, the organ was explanted. The subcutaneous islet grafts were excised with a margin of surrounding skin.

### 4.15 Intraperitoneal glucose tolerance test

An IPGTT was performed on day 28 or 101 after islet transplantation. For this purpose, the mice were i.p. injected with a 10% glucose solution after 16 h of fasting. Subsequently, blood glucose levels were determined at 0, 15, 30, 45, 60, 120, and 180 min using blood from the tail vein and analysed by a portable blood glucose monitoring system (GL50; Breuer).

To determine the total insulin content of the grafts, the islet transplants underneath the kidney capsule were dissected, lysed in 1 mL RIPA lysis buffer, and the intracellular insulin content was determined by an insulin ELISA kit according to the manufacturer's protocol.

### 4.16 Bioluminescence imaging

The visualization of luciferase activity was performed by means of bioluminescence imaging. After anaesthesia with 2% isoflurane in oxygen, mice were injected subcutaneously with 50 mg/kg IVISbrite d‐luciferin potassium salt bioluminescent substrate. After 5 min, the animals were imaged by means of an IVIS Spectrum In Vivo Imaging System (Perkin Elmer, Waltham, USA). The bioluminescence signal was detected by drawing a region of interest (ROI) over each graft. The total flux of the ROI was measured in photons/second (p/s) by means of the Living Image software (version 4.7.3; Perkin Elmer).

### 4.17 Immunohistochemistry

For the preparation of histological sections, mice were anaesthetised by an i.p. injection of ketamine (100 mg/kg body weight) and xylazine (12 mg/kg body weight) and euthanised by cervical dislocation. The kidney capsules or the subcutaneous tissue with the grafts were excised and fixed for 24 h in 4% paraformaldehyde (PFA). In addition, isolated islets were incubated for 45 min at 37°C in 100 μL HepatoQuick®, 50 μL human citrate plasma, and 10 μL 10% CaCl_2_ solution. The resulting clot was also fixed for 24 h in 4% PFA. The freshly generated nanofat was embedded in 1% agarose. The resulting clot was also fixed for 24 h in 4% PFA. The PFA‐fixed specimens were embedded in paraffin, and 3‐μm‐thick sections were cut.

The sections were stained with the indicated primary antibodies and visualised by their corresponding secondary antibodies. Cell nuclei were stained with Hoechst 33342 for fluorescence microscopy. HE as well as Sirius red stainings of individual sections were performed for bright field microscopy according to standard procedures. The sections were analysed by means of a BX60F microscope (Olympus, Hamburg, Germany).

### 4.18 Statistical analysis

All in vitro experiments were reproduced at least three times. The in vivo experiments were performed with five animals per group. After testing the data for normal distribution and equal variance, differences between two groups were assessed by the unpaired Student's *t*‐test. To test differences between multiple groups, one‐way ANOVA was applied. This was followed by the Tukey post hoc test. The statistical analysis was performed by means of Prism software 10.3.1 (GraphPad). The results were expressed as mean ± SEM. *p*‐values < 0.05 indicated statistical significance.

## AUTHOR CONTRIBUTIONS

*Conceptualization*: EA, SW, SR, MWL and LPR. *Methodology*: SW, AW, CBi, LSM, CW, CBe, EA, SR, LPR and BM. *Investigation*: SW, AW, CBi, LSM, CW, CBe, EA, SR and LPR, BM. *Visualization*: SW, AW, CBi, LSM, CW, CBe, EA and SR. *Project administration*: SW and EA. *Supervision*: EA, MWL and SR. *Writing – original draft*: EA, MDM, MWL and SW. *Writing – review and editing*: SW, AW, SR, BM, LPR, MDM, MWL and EA.

## FUNDING INFORMATION

This work was funded by the Deutsche Forschungsgemeinschaft (DFG, German Research Foundation)—411093008.

## CONFLICT OF INTEREST STATEMENT

The authors declare no conflicts of interest.

## PEER REVIEW

The peer review history for this article is available at https://www.webofscience.com/api/gateway/wos/peer‐review/10.1111/dom.70127.

## Supporting information


**Data S1.** Supporting Information.
**Figure S1.** (A) Quantitative analysis of body weights (g) of diabetic mice transplanted with SNF‐exposed islets or co‐transplanted with islets and the CNF at the indicated time points. On day 28, the grafts were removed by nephrectomy (marked by arrow). Diabetic mice transplanted with vehicle‐exposed islets served as positive control and nondiabetic mice served as negative control (*n* = 8). Mean ± SEM. (B) AUC (day −8–day 28) of the body weights shown in (A) (*n* = 8). Mean ± SEM.
**Figure S2.** HE stainings of grafts (marked by broken lines) under the kidney capsule of the different groups. Scale bar: 250 μm.
**Figure S3.** (A) Quantitative analysis of body weights (g) of diabetic mice transplanted with islets and nanofat. On day 101, the grafts were removed (marked by arrow). Diabetic mice transplanted with islets alone served as positive control and nondiabetic mice served as negative control (*n* = 6). Mean ± SEM. (B) AUC (day −8–day 101) of the body weights shown in (B). Mean ± SEM (*n* = 6).
**Figure S4.** HE staining of a subcutaneous graft from a mouse co‐transplanted with islets and nanofat on day 101. Scale bar: 100 μm.
**Figure S5.** (A–D) Bioluminescent images of a luciferase‐positive mouse (A), an isolated fatpad within a plate (B), nanofat in a well of a 96‐well plate (C) and nanofat in a 10 mL syringe (D). Scale bar: 10 mm.

## Data Availability

The data that support the findings of this study are available from the corresponding author upon reasonable request.
