# Supplementary figures and images for: Boosting the engraftment of subcutaneously transplanted pancreatic islets by nanofat
Source: Diabetes Obes Metab. 2025 Sep 24;27(12):7258–74. doi: 10.1111/dom.70127 (PMC12587261; doi:10.1111/dom.70127)

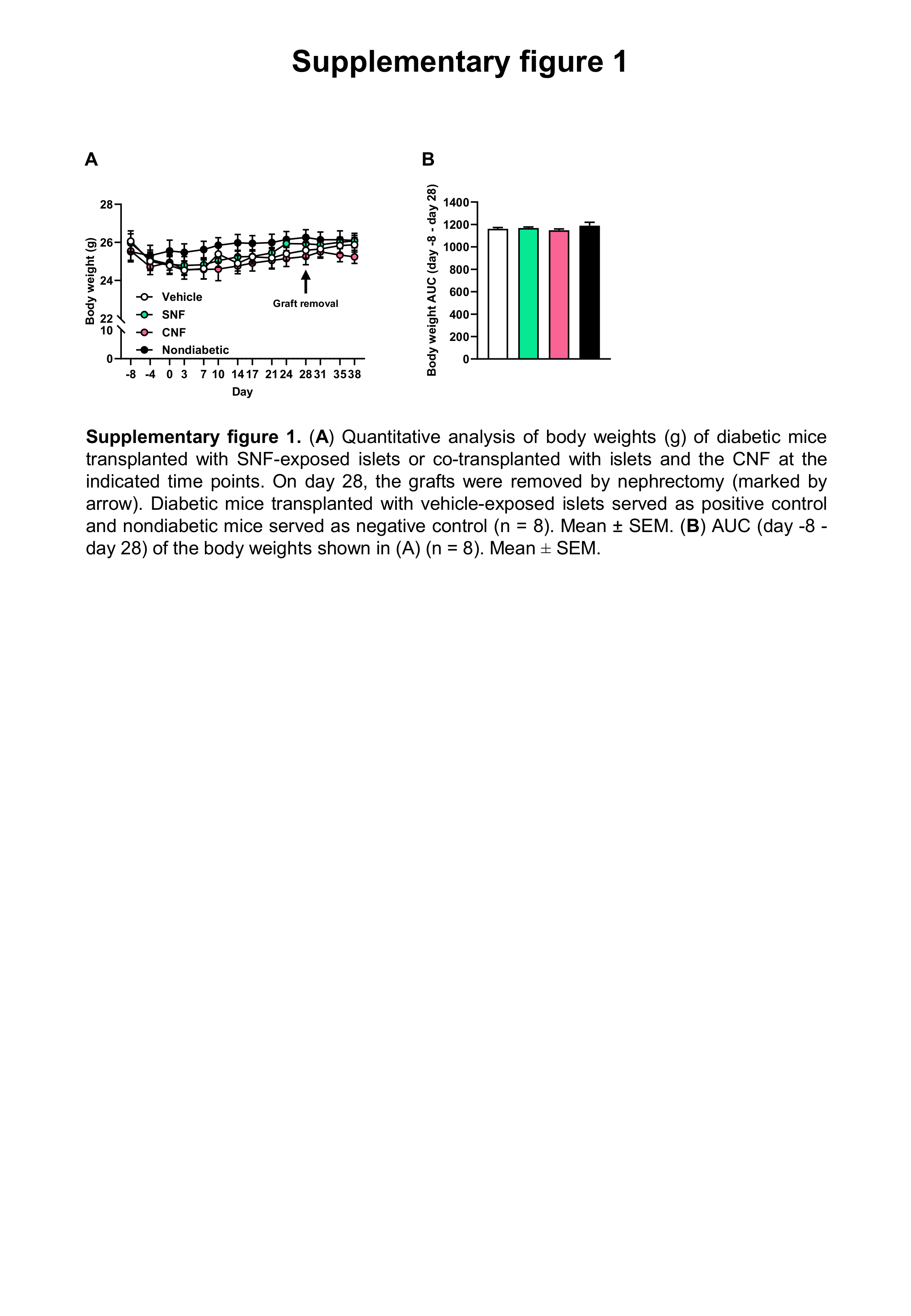

Supplement: Supplementary file 1 — Data S1. Supporting Information. Figure S1. (A) Quantitative analysis of body weights (g) of diabetic mice transplanted with SNF‐exposed islets or co‐transplanted with islets and the CNF at the indicated time points. On day 28, the grafts were removed by nephrectomy (marked by arrow). Diabetic mice transplanted with vehicle‐exposed islets served as positive control and nondiabetic mice served as negative control (n = 8). Mean ± SEM. (B) AUC (day −8–day 28) of the body weights shown in (A) (n = 8). Mean ± SEM. Figure S2. HE stainings of grafts (marked by broken lines) under the kidney capsule of the different groups. Scale bar: 250 μm. Figure S3. (A) Quantitative analysis of body weights (g) of diabetic mice transplanted with islets and nanofat. On day 101, the grafts were removed (marked by arrow). Diabetic mice transplanted with islets alone served as positive control and nondiabetic mice served as negative control (n = 6). Mean ± SEM. (B) AUC (day −8–day 101) of the body weights shown in (B). Mean ± SEM (n = 6). Figure S4. HE staining of a subcutaneous graft from a mouse co‐transplanted with islets and nanofat on day 101. Scale bar: 100 μm. Figure S5. (A–D) Bioluminescent images of a luciferase‐positive mouse (A), an isolated fatpad within a plate (B), nanofat in a well of a 96‐well plate (C) and nanofat in a 10 mL syringe (D). Scale bar: 10 mm. [file DOM-27-7258-s001.zip › dom70127-sup-0001-Supinfo1@dom-25-1915-op-File008.tif]

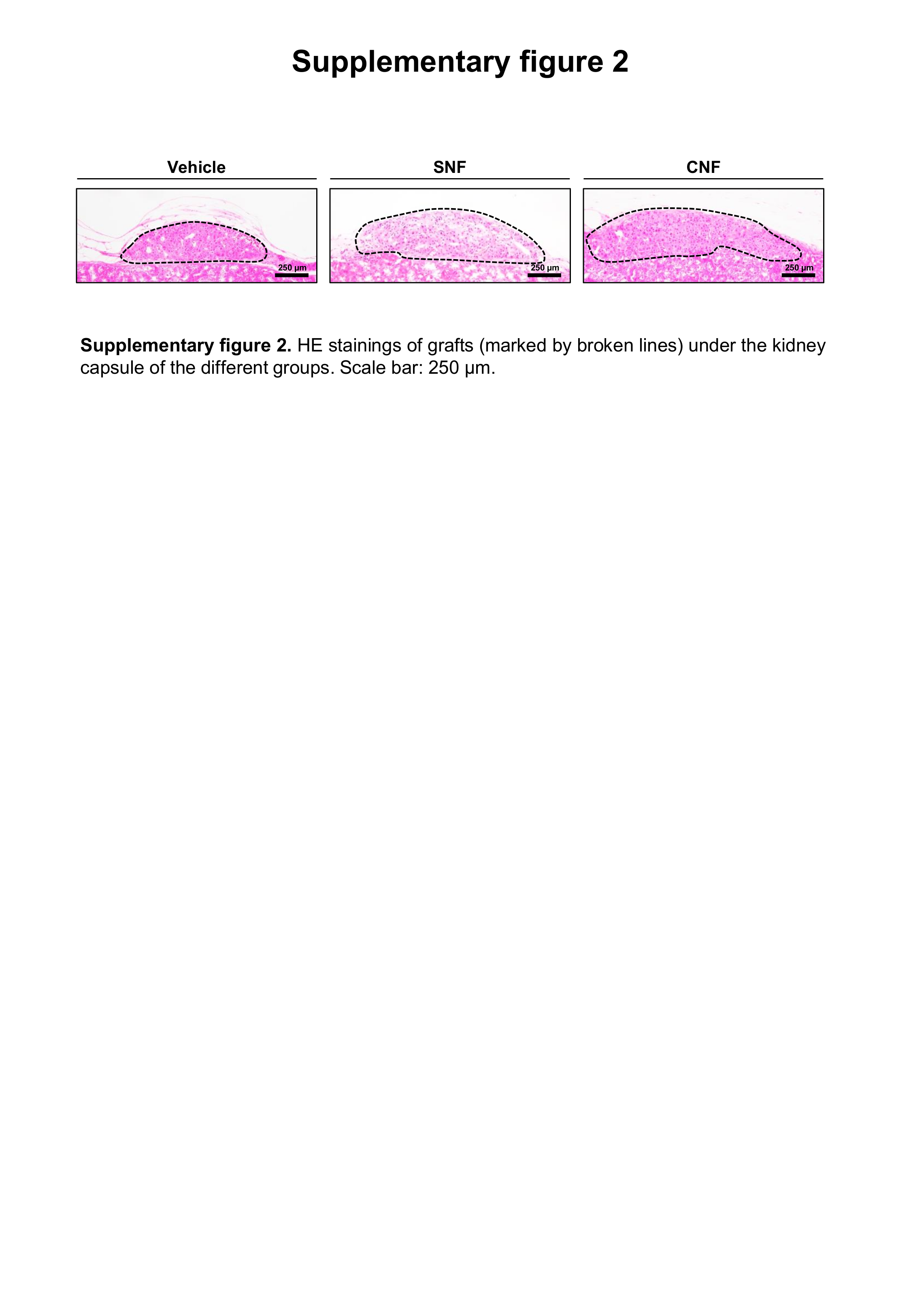

Supplement: Supplementary file 1 — Data S1. Supporting Information. Figure S1. (A) Quantitative analysis of body weights (g) of diabetic mice transplanted with SNF‐exposed islets or co‐transplanted with islets and the CNF at the indicated time points. On day 28, the grafts were removed by nephrectomy (marked by arrow). Diabetic mice transplanted with vehicle‐exposed islets served as positive control and nondiabetic mice served as negative control (n = 8). Mean ± SEM. (B) AUC (day −8–day 28) of the body weights shown in (A) (n = 8). Mean ± SEM. Figure S2. HE stainings of grafts (marked by broken lines) under the kidney capsule of the different groups. Scale bar: 250 μm. Figure S3. (A) Quantitative analysis of body weights (g) of diabetic mice transplanted with islets and nanofat. On day 101, the grafts were removed (marked by arrow). Diabetic mice transplanted with islets alone served as positive control and nondiabetic mice served as negative control (n = 6). Mean ± SEM. (B) AUC (day −8–day 101) of the body weights shown in (B). Mean ± SEM (n = 6). Figure S4. HE staining of a subcutaneous graft from a mouse co‐transplanted with islets and nanofat on day 101. Scale bar: 100 μm. Figure S5. (A–D) Bioluminescent images of a luciferase‐positive mouse (A), an isolated fatpad within a plate (B), nanofat in a well of a 96‐well plate (C) and nanofat in a 10 mL syringe (D). Scale bar: 10 mm. [file DOM-27-7258-s001.zip › dom70127-sup-0002-Supinfo2@dom-25-1915-op-File009.tif]

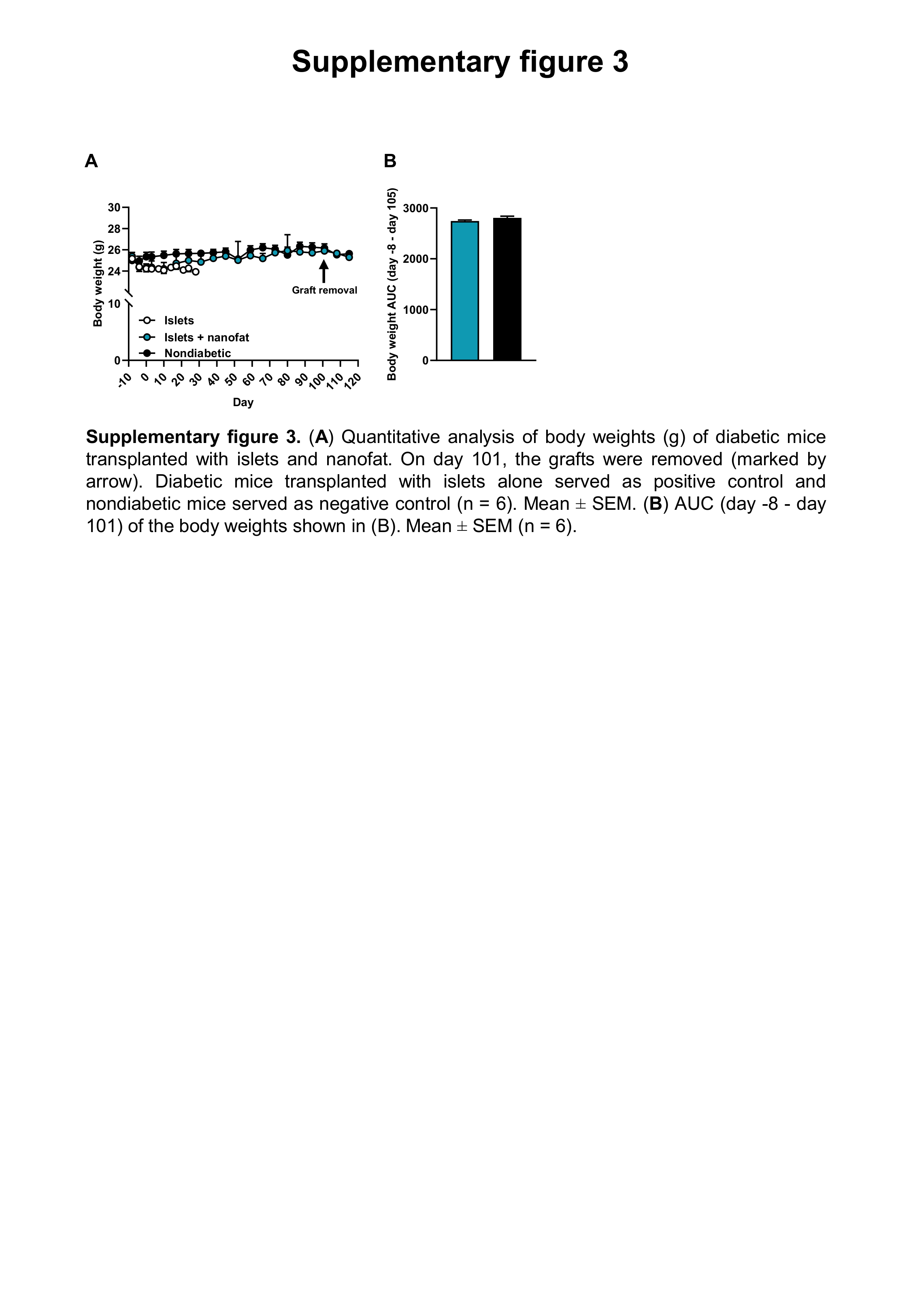

Supplement: Supplementary file 1 — Data S1. Supporting Information. Figure S1. (A) Quantitative analysis of body weights (g) of diabetic mice transplanted with SNF‐exposed islets or co‐transplanted with islets and the CNF at the indicated time points. On day 28, the grafts were removed by nephrectomy (marked by arrow). Diabetic mice transplanted with vehicle‐exposed islets served as positive control and nondiabetic mice served as negative control (n = 8). Mean ± SEM. (B) AUC (day −8–day 28) of the body weights shown in (A) (n = 8). Mean ± SEM. Figure S2. HE stainings of grafts (marked by broken lines) under the kidney capsule of the different groups. Scale bar: 250 μm. Figure S3. (A) Quantitative analysis of body weights (g) of diabetic mice transplanted with islets and nanofat. On day 101, the grafts were removed (marked by arrow). Diabetic mice transplanted with islets alone served as positive control and nondiabetic mice served as negative control (n = 6). Mean ± SEM. (B) AUC (day −8–day 101) of the body weights shown in (B). Mean ± SEM (n = 6). Figure S4. HE staining of a subcutaneous graft from a mouse co‐transplanted with islets and nanofat on day 101. Scale bar: 100 μm. Figure S5. (A–D) Bioluminescent images of a luciferase‐positive mouse (A), an isolated fatpad within a plate (B), nanofat in a well of a 96‐well plate (C) and nanofat in a 10 mL syringe (D). Scale bar: 10 mm. [file DOM-27-7258-s001.zip › dom70127-sup-0003-Supinfo3@dom-25-1915-op-File010.tif]

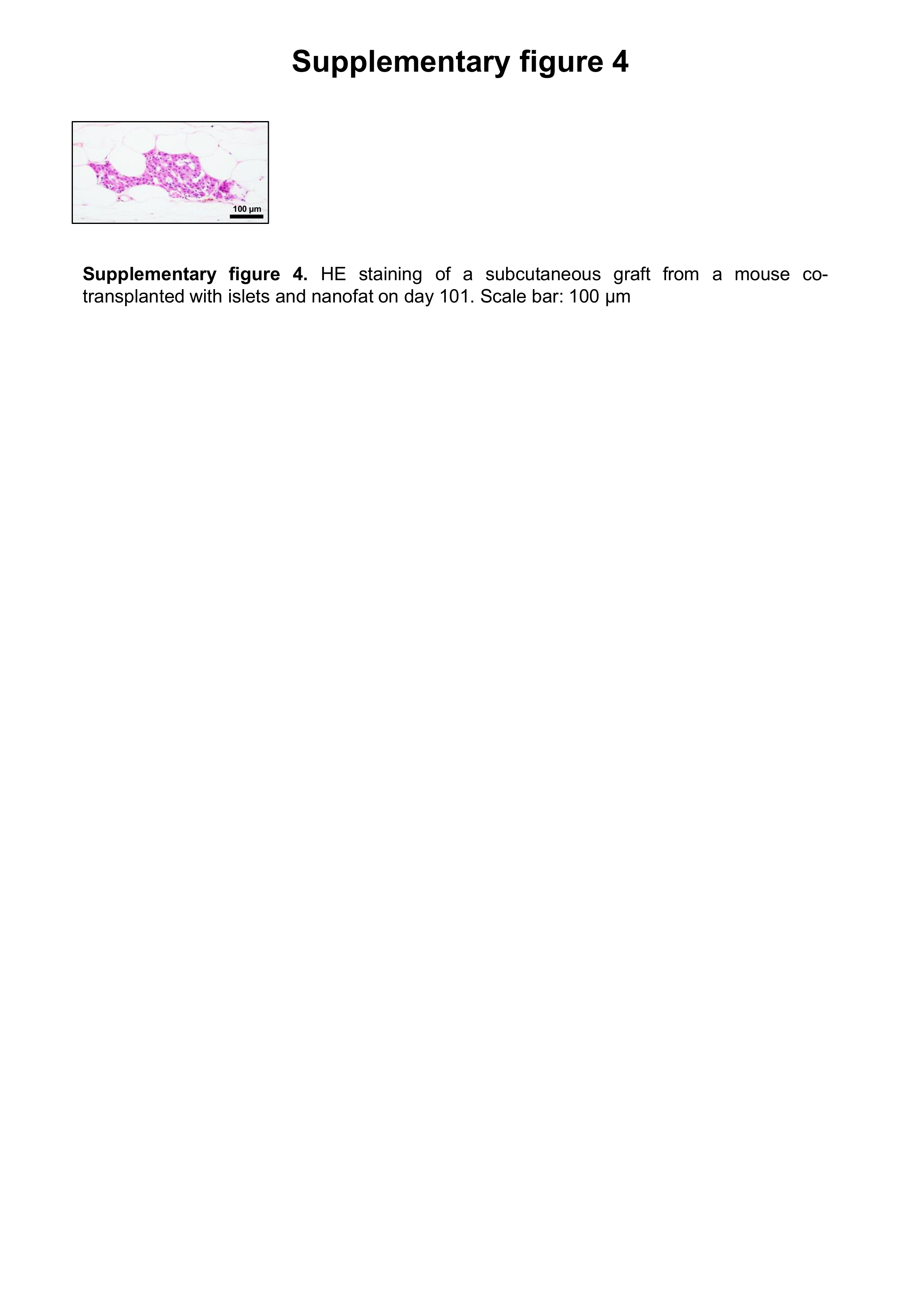

Supplement: Supplementary file 1 — Data S1. Supporting Information. Figure S1. (A) Quantitative analysis of body weights (g) of diabetic mice transplanted with SNF‐exposed islets or co‐transplanted with islets and the CNF at the indicated time points. On day 28, the grafts were removed by nephrectomy (marked by arrow). Diabetic mice transplanted with vehicle‐exposed islets served as positive control and nondiabetic mice served as negative control (n = 8). Mean ± SEM. (B) AUC (day −8–day 28) of the body weights shown in (A) (n = 8). Mean ± SEM. Figure S2. HE stainings of grafts (marked by broken lines) under the kidney capsule of the different groups. Scale bar: 250 μm. Figure S3. (A) Quantitative analysis of body weights (g) of diabetic mice transplanted with islets and nanofat. On day 101, the grafts were removed (marked by arrow). Diabetic mice transplanted with islets alone served as positive control and nondiabetic mice served as negative control (n = 6). Mean ± SEM. (B) AUC (day −8–day 101) of the body weights shown in (B). Mean ± SEM (n = 6). Figure S4. HE staining of a subcutaneous graft from a mouse co‐transplanted with islets and nanofat on day 101. Scale bar: 100 μm. Figure S5. (A–D) Bioluminescent images of a luciferase‐positive mouse (A), an isolated fatpad within a plate (B), nanofat in a well of a 96‐well plate (C) and nanofat in a 10 mL syringe (D). Scale bar: 10 mm. [file DOM-27-7258-s001.zip › dom70127-sup-0004-Supinfo4@dom-25-1915-op-File011.tif]

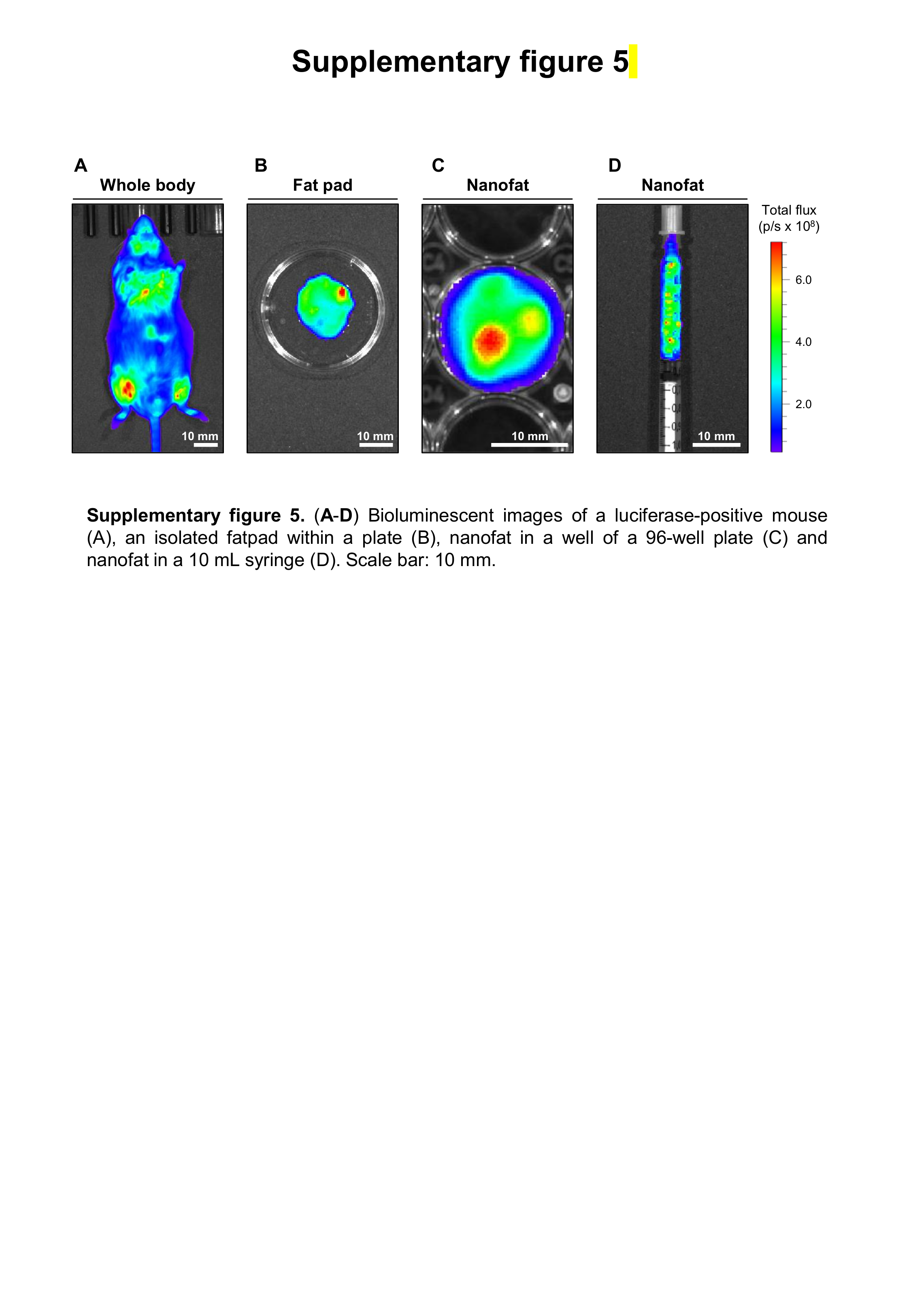

Supplement: Supplementary file 1 — Data S1. Supporting Information. Figure S1. (A) Quantitative analysis of body weights (g) of diabetic mice transplanted with SNF‐exposed islets or co‐transplanted with islets and the CNF at the indicated time points. On day 28, the grafts were removed by nephrectomy (marked by arrow). Diabetic mice transplanted with vehicle‐exposed islets served as positive control and nondiabetic mice served as negative control (n = 8). Mean ± SEM. (B) AUC (day −8–day 28) of the body weights shown in (A) (n = 8). Mean ± SEM. Figure S2. HE stainings of grafts (marked by broken lines) under the kidney capsule of the different groups. Scale bar: 250 μm. Figure S3. (A) Quantitative analysis of body weights (g) of diabetic mice transplanted with islets and nanofat. On day 101, the grafts were removed (marked by arrow). Diabetic mice transplanted with islets alone served as positive control and nondiabetic mice served as negative control (n = 6). Mean ± SEM. (B) AUC (day −8–day 101) of the body weights shown in (B). Mean ± SEM (n = 6). Figure S4. HE staining of a subcutaneous graft from a mouse co‐transplanted with islets and nanofat on day 101. Scale bar: 100 μm. Figure S5. (A–D) Bioluminescent images of a luciferase‐positive mouse (A), an isolated fatpad within a plate (B), nanofat in a well of a 96‐well plate (C) and nanofat in a 10 mL syringe (D). Scale bar: 10 mm. [file DOM-27-7258-s001.zip › dom70127-sup-0005-Supinfo5@dom-25-1915-op-File012.tif]
